# Obesity and Polypharmacy among African American Older Adults: Gender as the Moderator and Multimorbidity as the Mediator

**DOI:** 10.3390/ijerph16122181

**Published:** 2019-06-20

**Authors:** Shervin Assari, Cheryl Wisseh, Mohsen Bazargan

**Affiliations:** 1 Department of Family Medicine, Charles R Drew University of Medicine and Science, Los Angeles, CA 90095, USA; mobazarg@cdrewu.edu; 2 Department of Pharmacy Practice, West Coast University School of Pharmacy, Los Angeles, CA 91606, USA; cWisseh@westcoastuniversity.edu; 3 Department of Family Medicine, University of California Los Angeles (UCLA), Los Angeles, CA 90095, USA

**Keywords:** African American, Black, elderly, older adults, medication use, polypharmacy, body mass index, obesity

## Abstract

Despite high prevalence of obesity and polypharmacy among African American (AA) older adults, little information exists on the associations between the two in this population. This study explored the association between obesity and polypharmacy among AA older adults who were residing in poor urban areas of South Los Angeles. We also investigated role of gender as the moderator and multimorbidity as the mediator of this association. In a community-based study in South Los Angeles, 308 AA older adults (age ≥ 55 years) were entered into this study. From this number, 112 (36.4%) were AA men and 196 (63.6%) were AA women. Polypharmacy (taking 5+ medications) was the dependent variable, obesity was the independent variable, gender was the moderator, and multimorbidity (number of chronic medical conditions) was the mediator. Age, educational attainment, financial difficulty (difficulty paying bills, etc.), income, marital status, self-rated health (SRH), and depression were the covariates. Logistic regressions were used for data analyses. In the absence of multimorbidity in the model, obesity was associated with higher odds of polypharmacy in the pooled sample. This association was not significant when we controlled for multimorbidity, suggesting that multimorbidity mediates the obesity-polypharmacy link. We found significant association between obesity and polypharmacy in AA women not AA men, suggesting that gender moderates such association. AA older women with obesity are at a higher risk of polypharmacy, an association which is mainly due to multimorbidity. There is a need for screening for inappropriate polypharmacy in AA older women with obesity and associated multimorbidity.

## 1. Introduction

Polypharmacy (taking 5+ medications a day) [1,2] increases the risks of adverse drug events (ADEs), drug–drug interactions, and medication nonadherence [3,4]. Polypharmacy also increases the risk of various undesired but preventable health outcomes [5,6,7,8], including, but not limited to, cognitive decline, falls, emergency department visits, unnecessary hospitalization, and mortality [3,4,9,10]. Polypharmacy also adds to the risk of inappropriate use of medications (IUM) [11,12]. Such negative health outcomes impose a risk on the patient and an economic burden to the health care system [11]. 

There is a need to study the association between obesity and polypharmacy in African American (AA) older adults for multiple reasons. First, there is a growing epidemic of polypharmacy in the US as the population is aging [11,12,13,14]. Given that advanced age is the largest contributing factor to the prevalence of polypharmacy, we should know more about the correlates of polypharmacy in AA older adults who have multiple chronic diseases and multimorbidity. While it has been demonstrated that polypharmacy is closely associated with major health problems [9,10], little is known on how obesity may contribute to the risk of polypharmacy of AA older adults. Inappropriate polypharmacy is dangerous for older adults who experience vulnerability due to age-related physiological changes and frailty [15]. Some reports suggest that half of older adults take at least one medication that is unnecessary [11], which increases their risk of ADEs and drug–drug interactions as well as health care costs [11,16,17]. Inappropriate polypharmacy is also responsible for up to 12% of all largely preventable hospitalizations of elderly individuals [18,19,20]. 

When compared to obesity, more is known regarding the associations of polypharmacy with other health problems such as multimorbidity, chronic diseases (CDs) [21], self-rated health (SRH), depression [22,23,24], and distress [25]. From the existing literature, few studies have enrolled AA adults [2], and even fewer have specifically focused on AA older adults [25]. As race alters correlates of several health constructs [26], including, but not limited to, obesity [27,28,29,30,31], additional studies are required that explore the association between polypharmacy and obesity in AA older adults. Such studies may generate results that can potentially help with health promotion programs and resources targeting the reduction of inappropriate polypharmacy in AA older adults. 

There is a paucity of data on whether obesity correlates with polypharmacy among AA older adults [2,25,32,33]. Furthermore, although some recent studies have explored correlates of polypharmacy in this population [34,35,36,37], very limited knowledge is available on how elimination of disparities in obesity would contribute to the elimination of disparities in the polypharmacy in AA older adults in US.

Several potential factors increase vulnerability of AA older adults to polypharmacy. Low socioeconomic status, financial difficulties, low health literacy, multiple competing health needs, and poor access to healthcare (% uninsured and underinsured) are all crucial components of the recipe to polypharmacy and its associated burden [15]. In comparison to their White counterparts, AA older adults have a lower likelihood of receiving the most effective, and the most up to date medication regimens for their health conditions [38,39]. Lower quality of care combined with a wide range of biases against AA older adults who attend the healthcare system increases their risk of being prescribed multiple medication regimens [2,25]. As a result, racial disparities may exist in the risk of inappropriate medication use and polypharmacy among older adults [38,39]. 

Gender is a central risk factor that shapes prevalence of polypharmacy [40,41]. There are multiple studies showing that women may have a higher risk of polypharmacy than men [40,41,42,43]. In another study of AA older adults, gender, in addition to multimorbidity, potentially inappropriate medication use, and number of healthcare providers, predicted polypharmacy [25]. As gender alters determinants and consequences of various health outcomes [40,42] including but not limited to polypharmacy [44,45,46,47], additional studies should explore the link between obesity and polypharmacy in AA older adults as well as other factors such chronic disease burden and gender.

### Aims

The purpose of the current study was to investigate the associations between obesity and polypharmacy (taking 5+ medications) among AA older adults. We also tested whether gender moderates and whether multimorbidity (number of CDs) mediates this association. 

## 2. Materials and Methods

### 2.1. Design and Setting

The current cross-sectional study used a survey and a comprehensive evaluation of medications taken. The study was conducted in south Los Angeles, California, USA. Data were gathered between 2015 and 2018 [48,49]. 

### 2.2. Ethics

The study protocol received ethical approval by the Charles R. Drew University of Medicine and Science (CDU) Institutional Review Board (IRB), Los Angeles, California, USA (CDU IRB#: 14-12-2450-05). All participants provided a written informed consent before they were enrolled to the study. Participants were financially compensated.

### 2.3. Process and Data Collection 

The data collection was composed of two main components: (1) a structured face-to-face interview and (2) a comprehensive investigation of medications taken. During the structured interviews, a wide range of data were collected that included (1) demographic factors (gender and age), (2) SES (educational attainment, financial difficulty, income, marital status, or living alone), and (3) health including but not limited to multimorbidity (number of CDs), SRH, depression, and obesity.

### 2.4. Participants

Our study enrolled a nonrandom sample of AA older adults from economically disadvantaged areas in South Los Angeles (e.g., Watts area). Using convenience sampling, participants were eligible for enrollment if they were (1) African American/AA, (2) were 55 years or older, and (3) could complete an interview in English. Participants were excluded if they were (1) institutionalized or (2) enrolled in other clinical trials. 

Participants in our study were sampled from 11 senior housing apartment units, 16 AA churches, and several low-income public housing projects, all located in Service Planning Area 6 (SPA 6), Los Angeles County, California. The LA Department of Public Health divided LA County into eight SPAs to better conduct surveillance and to better provide public health services to LA county residents, considering their specific needs. In SPA 6, 49% of older adults are AAs, 58% of adults have an income level less than 200% of the federal poverty line (FPL), and 36% are uninsured. Between 2013 and 2015, homeless AA individuals in SPA6 increased from 39% to 70% [48,49]. 

### 2.5. Analytical Sample

The current analysis included 308 AA older adults (age ≥ 55 years) which comprised of 112 (36.4%) AA men and 196 (63.6%) AA women.

### 2.6. Measurements 

Obesity: This study measured BMI based on measured weight and height. Height was measured in feet/inches. Body weight was measured in pounds. To calculate BMI, first, height, and weight were converted to meters and kilograms, respectively. We calculated BMI by dividing body weight (kilograms) by height (meters squared). Obesity was defined as body mass index (BMI) equal to or more than 30.

Polypharmacy: In this study, polypharmacy was measured based on a comprehensive evaluation of all medications of the individual. Polypharmacy was defined as use of 5+ medications in a single day [1]. 

Sociodemographic covariates: Age, SES (educational attainment, financial difficulty, income, marital status, and living alone) were the sociodemographic variables. Age and educational attainment (years of schooling) were continuous variables. Income was monthly income in US dollars and financial difficulties were measured using three items that were based on Pearlin’s list of main chronic financial difficulties experienced by low income individuals [50,51]. The three items were the frequency by which a participant did not have enough money to afford (1) clothing, (2) food, and (3) paying bills. Responses ranged from 1 (never) to 5 (always). We calculated a sum score (a higher score reflecting more financial difficulties). (Cronbach’s alpha = 0.92).

Depression: This study used the 15-item short Geriatric Depression Scale (GDS) to evaluate depression [26]. Responses were on a “yes” or “no” scale. A summary score was calculated with a potential range between 0 and 15. A higher score indicated more depression. The GDS-short form has excellent reliability and validity and has been extensively used to measure depression among older adults in both clinical and community settings [49,50,51,52,53].

Self-Rated Health: We asked participants about their overall health. The responses ranged from excellent (1) to poor (5) [54]. We treated SRH as a continuous variable with a range from 1 to 5, where a higher score reflects poorer health. Poor SRH in turn, predicts all-cause mortality in the general population [55,56] as well as patients with chronic disease [56]. Review articles and multiple original studies have established high predictive validity of poor SRH as a robust determinant of mortality risk, above and beyond covariates such as SES and health [56,57,58].

Mediator: Participants’ multimorbidity was operationalized as the number of CDs a participant had. Participants were asked about the presence of the following eleven CDs: hypertension, heart disease, diabetes, lipid disorder/hypercholesterolemia, cancer, asthma, osteoarthritis, thyroid disorder, chronic obstructive pulmonary disease, rheumatoid arthritis, or gastrointestinal disease. Participants reported if a physician had ever told them that they have any of the CDs previously listed. Multimorbidity was operationalized as a sum score that reflected number of CDs that were present. Self-reported CDs have high validity [59,60,61]. 

Moderator: Gender was treated as a dichotomous variable (1 woman, 0 men).

### 2.7. Data Analysis

For data analysis, we used SPSS 23.0 (IBM, Armonk, New York, NY, USA). We reported mean, standard deviation (SD), frequency (*n*), and relative frequency (%) to describe our participants both in the pooled sample and each gender. We used Chi-square and independent samples *t*-test for bivariate analyses. For multivariable analysis, logistic regression models were used. In our logistic models, polypharmacy was the main dependent variable, obesity was the main independent variable, and demographic factors (age) and SES (educational attainment, financial difficulty, income, marital status, and living alone), and SRH and depression were the covariates. Gender was the moderator and multimorbidity (number of CDs) was the mediator. Model 1 and Model 2 were conducted in the pooled sample. Model 3 to Model 4 were conducted in each gender. Models were different in regards of controlling or not controlling for multimorbidity. Mediation and moderation were defined based on Baron and Kenny [29]. We defined a third variable as a moderator if it changed the association between two variables. We defined a 3rd variable a mediator if it was associated with both of our variables and its inclusion as a control variable fully explained our primary association of interest [29]. We ran models in the pooled sample by gender and results were reported as odds ratios (OR), standard errors (SE), and *p*-values. 

## 3. Results

### 3.1. Descriptive Statistics

Table 1 describes the pooled sample based on gender. The sample included 308 AA older adults (age ≥ 55 years). From this number, 112 were AA men (36.4%) and 196 were AA women (63.6%). Overall, approximately 44.8% and 68.5% had obesity and polypharmacy, respectively. 

Table 1 also compares older AA men and older AA women for the study variables. Older AA men were significantly younger than older AA women. Older AA women reported more financial difficulties than older AA men. Older AA women also reported higher multimorbidity (number of CDs) compared to older AA men. Polypharmacy, obesity, and poor SRH were also more common in older AA women than older AA men (Table 1).

### 3.2. Bivariate Correlations

Table 2 shows three correlation matrices: one in the pooled sample, one in older AA men, and one in older AA women. There was a positive correlation between multimorbidity (number of CDs) and polypharmacy in the pooled sample, older AA women, and older AA men. There was also a positive correlation between obesity and polypharmacy in the pooled sample and in older AA women but not older AA men (Table 2).

### 3.3. Multivariable Models

As Table 3 shows, obesity was associated with polypharmacy in the pooled sample, when multimorbidity was not adjusted in the model. Obesity was no longer associated with polypharmacy in the pooled sample, however, after adjusting for multimorbidity. This suggests that multimorbidity is the mechanism by which obesity is associated with polypharmacy in the pooled sample. 

### 3.4. Multivariable Models

As Table 4 demonstrates, gender differences were found in the association between obesity and polypharmacy. The association between obesity and polypharmacy was significant for older AA women but not older AA men. For older AA women, obesity was associated with polypharmacy, when multimorbidity was not controlled in the model. For AA women, obesity was no longer associated with polypharmacy after controlling for multimorbidity. This suggests that multimorbidity is the mechanism by which obesity is associated with polypharmacy for older AA women (Table 4).

## 4. Discussion

This study revealed three findings: first, obesity was associated with polypharmacy in AA older adults, above and beyond confounders. Second, the association between obesity and polypharmacy was moderated by gender. Obesity is associated with polypharmacy in older AA women but not in older AA men. Third, the association between obesity and polypharmacy was mediated by multimorbidity (number of CDs).

AA older adults with obesity, particularly women, were more likely to have polypharmacy. Previous research has shown that AA older adults with polypharmacy also report multimorbidity, worse SRH, in addition to depression and psychological distress [25]. In a study of AA adults older than 18 years old, polypharmacy increased the risk of mental distress. Furthermore, in that study, the association between polypharmacy and psychological distress was net of gender and all confounders such as SES and multimorbidity [47]. Other studies have shown that polypharmacy is associated with depression and poor SRH [47]. The current finding adds to the literature and suggests that polypharmacy is also associated with obesity, an association which is due to multimorbidity. Additionally, this information adds to the literature that exists on the link between polypharmacy, depression, distress, and SRH [25,47].

In the current study, gender was a moderator of the association between obesity and polypharmacy. In a recent study, gender changed the association between polypharmacy and SRH [47]. More specifically, poor SRH was indicative of higher odds of polypharmacy in older AA women but not older AA men. Gender differences in correlates of obesity in AAs is well-known and obesity is differently associated with SES, depression, and other factors in male and female AAs across age groups, compared to White people [62,63,64,65,66,67]. 

Gender impacts both prevalence and correlates of obesity and polypharmacy. Compared to men, women are at higher risk of polypharmacy [42,43]. Even with a lower risk of fatal chronic medical conditions, women are more likely than men to feel worse health [63]. Although some studies have failed to show any gender differences in polypharmacy [64], most of previous studies have documented a higher prevalence of polypharmacy in women than men [42,43,44]. Women may also be at a higher risk of potentially inappropriate medication use [14,42,43]. Due to gendered socialization [65], women are more likely than men to seek help for and communicate about their symptoms. This likely increases the chance of having multiple comorbid medical conditions [66]. At each level of multiple chronic medical conditions, women are more likely than men to seek professional care [65]. Women are more likely to be aware of their health problems and symptoms [63], they have more effective communication skills to share their symptoms with their health care providers. Therefore, there is a tendency for women to have an increased likelihood to be diagnosed with multiple chronic conditions. For some men, masculine ideologies operate as a barrier against seeking healthcare for their health problems, so they are less likely to be diagnosed with chronic disease [68,69,70,71,72,73]. As a result, men do not effectively share their symptoms with their health care providers [71], which causes a substantial delay in how and when they are diagnosed with a disease [72]. As a result, gender differences emerge in polypharmacy [42]. These processes have implications for emerging gender differences in correlates of polypharmacy in men and women. 

The results of this study are not generalizable to the US population because the sample is not a representative sample. The current sample is composed of economically disadvantaged residents that live in poor urban areas. This is important given the protective effect of high SES against polypharmacy [14,74,75,76] and obesity [27,28,29,77,78]. It is well known that that SES shapes the risk of polypharmacy in AA men and women [14,74,75,76]. This is further corroborated by the fact that underserved AA older adults experience high levels of financial difficulty, experience struggles accessing the health care system, and have high numbers of chronic diseases. 

Polypharmacy, multimorbidity, poor health, and obesity are common and interrelated issues among AA older men and women [79,80,81,82,83]. As all of these conditions are risk factors for morbidity and mortality [84,85,86,87,88,89], these factors should be addressed jointly. Health promotion programs that address AA older adults in underserved communities should consider the link between obesity and polypharmacy. More specifically, programs and interventions that wish to promote health toward AA older adults in underserved communities should address polypharmacy, multimorbidity, and obesity as common elements of morbidity and mortality of AA older adults. Studies that address medication challenges of the AA community should also not discount or overlook obesity given the close link between obesity, multimorbidity, and polypharmacy.

The link between polypharmacy and BMI may be because of multimorbidity. Future research should address if prevention of obesity will be an effective way to reduce inappropriate polypharmacy for AA older adults [84,86]. We are not aware of many evidence-based interventions that are available to reduce risk of obesity or polypharmacy among AA older adults [90,91]. As shown in existing systematic reviews, the effect size of existing interventions that address inappropriate polypharmacy is small [92]. We should develop, implement, and evaluate new interventions that reduce unnecessary polypharmacy and associated morbidity for AA older adults [93,94]. 

Our models 1 to 3 showed that ‘living alone’ had a large OR which was statistically significant. Thus, AA older adults living alone are far more likely to use polypharmacy than their counterparts who are not living alone. The mechanism of this finding is not known, and necessitates additional research. Literature has connected social isolation and lack of social support to a wide range of health problems such as depression, chronic disease, and poor self-rated health [95,96,97,98,99]. This finding is important because social isolation is common among AA older adults [96,99]. 

The results shown by this study may inform public health programs as well as clinical practice to promote the health and well-being of economically disadvantaged AA older adults in urban communities. We argue that the health promotion of AA older adults should address polypharmacy. 

Knowing that gender alters the correlates of polypharmacy, interventions that wish to address medication related challenges of AA older adults may benefit from the gender of the participant. Tailoring such programs to the gender of the participant may help with enhancing the efficacy of such interventions. Future research is needed to understand how addressing medication-related challenges can improve the health and wellbeing of AA older adults in underserved communities.

### Limitations

Our study has a few limitations. As a cross-sectional design, we cannot make any causal inferences. This study only measured the quantity rather than the classification and indication of medications taken. Without knowing such information regarding participants’ medications, it is impossible to know the true percentage of participants that are receiving inappropriate polypharmacy. Future work should also measure drug-drug, drug-disease, and drug-consumption interactions in the population. In addition, we did not verify the self-reported multimorbidity using other sources such as medical claims, medical charts, or pharmacy data. Some potentially important confounders were not included. For example, we did not measure psychiatric problems or somatization. In addition, all our variables were measured at an individual level. We also did not collect data on disability, activities of daily living (ADLs), cognition, and frailty. The sample was not random but a convenient sample of AA older adults in South Los Angeles [25,36,47,48,49,67]. As a such, replication studies are needed using random samples that generate generalizable findings. In addition, most of our participants were AA women which is another reason our findings are not generalizable to the US AA population. This study only included AA older adults. Future comparative studies regarding various ethnic groups and their corresponding levels of obesity and polypharmacy are still needed. Theoretically, the strength of the link between obesity and polypharmacy may change across population subgroups, and obesity may be a stronger risk factor for polypharmacy in some versus other ethnic groups. Lack of information on the type of medications is a major limitation to this study. As such, drug-drug interactions, and the extent to which polypharmacy was inappropriate could not be evaluated. Given these limitations, the results of our study results are preliminary, however, they still may contribute to improvement of the health of economically challenged older AAs. The result contributes to how obesity may increase risk of polypharmacy among AA older adults.

## 5. Conclusions

In summary, among AA older adults, particularly for women, obesity is associated with polypharmacy, and this association is mainly due to higher risk of multimorbidity in obese people. Given the overlaps between obesity, multimorbidity, and polypharmacy interventions that address health of obese older AA women may include screening for inappropriate polypharmacy.

## Figures and Tables

**Table 1:** Descriptive characteristics in the pooled sample and by gender.

Characteristics	All		Men		Women	
	*n* (%)		*n* (%)		*n* (%)	
Gender						
Men	112	36.4	112	100.0	-	-
Women	196	63.6	-	-	196	100.0
Living Alone						
No	282	91.6	98	87.5	184	93.9
Yes	26	8.4	14	12.5	12	6.1
Marital Status (Married) *						
No	86	27.9	38	33.9	48	24.5
Yes	222	72.1	74	66.1	148	75.5
Poor SRH *						
No	176	57.3	38	33.9	48	24.5
Yes	131	42.7	74	66.1	148	75.5
Obese *						
No	170	55.2	79	70.5	91	46.4
Yes	138	44.8	33	29.5	105	53.6
Polypharmacy *						
No	97	31.5	42	37.5	55	28.1
Yes	211	68.5	70	62.5	141	71.9
Age (Years) *	69.74	9.18	68.30	8.46	70.57	9.49
Educational Attainment	12.72	2.12	12.43	2.36	12.88	1.95
Financial Difficulty *	11.82	6.04	13.15	6.61	11.06	5.55
Income	2.69	1.11	2.84	1.32	2.61	0.97
Depression	3.26	3.03	3.42	2.82	3.17	3.15
Multimorbidity (Number of CDs) *	4.32	1.84	3.91	1.83	4.55	1.81

CDs: Chronic Diseases; SRH: Self-Rated Health. * *p* < 0.05. (Chi-square or independent *t*-test).

**Table 2:** Bivariate associations between study variables overall and by gender.

	1	2	3	4	5	6	7	8	9	10	11	12
**All**												
1 Gender (Women)	1	0.119 *	0.103	−0.167 **	−0.100	−0.110	0.101	−0.044	−0.040	0.233 **	0.098	0.169 **
2 Age (Years)		1	−0.171 **	−0.272 **	0.029	−0.092	0.192 **	−0.155 **	−0.207 **	−0.086	0.158 **	0.070
3 Educational Attainment			1	−0.122 *	0.215 **	0.074	−0.097	−0.121 *	−0.162 **	−0.022	−0.068	−0.090
4 Financial Difficulty				1	−0.154 **	0.044	−0.024	0.206 **	0.443 **	0.037	−0.102	0.141 *
5 Monthly Income					1	0.244 **	−0.214 **	−0.071	−0.127 *	0.090	−0.101	−0.164 **
6 Marital Status (Married)						1	−0.384 **	−0.026	0.055	0.032	0.030	0.062
7 Living Alone							1	−0.019	−0.023	0.022	0.201 **	0.128
8 SRH (Poor)								1	0.383 **	0.087	0.062	0.198 **
9 Depression									1	0.089	0.049	0.242 **
10 Obesity										1	0.119 *	0.147 **
11 Polypharmacy											1	0.374 **
12 Multimorbidity (Number of CDs)												1
**Men**												
1 Gender (Women)	-	-	-	-	-	-	-	-	-	-	-	-
2 Age (Years)		1	−0.333 **	−0.279 **	0.051	−0.177	0.207 *	−0.180	−0.080	−0.149	0.030	0.059
3 Educational Attainment			1	−0.214 *	0.267 **	0.172	−0.271 **	−0.136	−0.253 **	−0.060	−0.102	−0.181
4 Financial Difficulty				1	−0.241 *	0.069	0.005	0.104	0.416 **	−0.024	−0.083	0.237 *
5 Monthly Income					1	0.192 *	−0.176	−0.095	−0.135	0.156	−0.068	−0.166
6 Marital Status (Married)						1	−0.470 **	−0.075	0.059	0.111	0.070	0.078
7 Living Alone							1	−0.026	−0.074	0.008	0.185	0.089
8 SRH (Poor)								1	0.240 *	0.078	0.005	0.084
9 Depression									1	0.113	−0.016	0.316 **
10 Obesity										1	0.056	0.010
11 Polypharmacy											1	0.398 **
12 Multimorbidity (Number of CDs)												1
**Women**												
1 Gender (Women)	-	-	-	-	-	-	-	-	-	-	-	-
2 Age (Years)		1	−0.098	−0.248 **	0.036	−0.015	0.169 *	−0.136	−0.261 **	−0.103	0.214 **	0.046
3 Educational Attainment			1	−0.019	0.193 **	0.004	0.014	−0.105	−0.104	−0.040	−0.061	−0.060
4 Financial Difficulty				1	−0.113	−0.014	−0.018	0.271 **	0.466 **	0.144 *	−0.091	0.131
5 Monthly Income					1	0.282 **	−0.233 **	−0.061	−0.134	0.094	−0.113	−0.141
6 Marital Status (Married)						1	−0.300 **	0.003	0.047	0.024	0.017	0.087
7 Living Alone							1	−0.007	0.012	−0.007	0.199 **	0.129
8 SRH (Poor)								1	0.457 **	0.111	0.106	0.281 **
9 Depression									1	0.096	0.092	0.220 **
10 Obesity										1	0.124	0.168 *
11 Polypharmacy											1	0.344 **
12 Multimorbidity (Number of CDs)												1

CDs: Chronic Diseases; SRH: Self-Rated Health. * *p* < 0.05. ** *p* < 0.001. (Pearson correlation test).

**Table 3:** Logistic regressions on the associations between gender, obesity, multimorbidity, and polypharmacy in the pooled sample.

Characteristics	OR	Std. Error	95% CI		Sig
*Model 1*					
Gender (Women)	1.14	0.29	0.65	2.01	0.639
Age (Years)	1.03	0.02	1.00	1.07	0.039
Educational Attainment	0.99	0.07	0.86	1.14	0.849
Financial Difficulty	0.95	0.03	0.90	0.99	0.030
Monthly Income	0.79	0.12	0.62	1.01	0.061
Marital Status (Married)	3.71	0.54	1.28	10.80	0.016
Living Alone	2.67	0.31	1.46	4.87	0.001
SRH (Poor)	1.37	0.29	0.77	2.44	0.281
Depression	1.07	0.05	0.96	1.19	0.203
Obesity	1.73	0.28	1.00	2.99	0.051
Constant	0.21	1.73			0.364
*Model 2*					
Gender (Women)	0.89	0.31	0.49	1.62	0.692
Age (Years)	1.03	0.02	0.99	1.06	0.129
Educational Attainment	0.99	0.08	0.86	1.15	0.940
Financial Difficulty	0.93	0.03	0.88	0.98	0.007
Monthly Income	0.85	0.13	0.66	1.10	0.212
Marital Status (Married)	2.72	0.60	0.85	8.78	0.093
Living Alone	2.41	0.33	1.26	4.61	0.008
SRH (Poor)	1.22	0.31	0.66	2.25	0.531
Depression	1.02	0.06	0.91	1.13	0.747
Obesity	1.48	0.30	0.83	2.64	0.188
Multimorbidity	1.67	0.10	1.38	2.02	0.000
Constant	0.07	1.85			0.158

CDs: Chronic Diseases; SRH: Self-Rated Health. Model 1: Obesity only. Model 2: Obesity and multimorbidity.

**Table 4:** Logistic regressions on the associations between obesity, multimorbidity, and polypharmacy in older AA men and older AA women.

Characteristics	OR	Std. Error	95% CI		Sig	OR	Std. Error	95% CI		Sig
	Men					Women				
*Model 3*										
Age (Years)	0.98	0.03	0.93	1.04	0.529	1.06	0.02	1.02	1.11	0.005
Educational Attainment	0.90	0.12	0.71	1.13	0.349	1.02	0.10	0.85	1.24	0.808
Financial Difficulty	0.94	0.04	0.87	1.02	0.131	0.93	0.04	0.87	1.00	0.039
Monthly Income	0.88	0.17	0.63	1.22	0.440	0.73	0.19	0.51	1.06	0.098
Marital Status (Married)	4.22	0.75	0.98	18.20	0.054	3.19	0.87	0.58	17.72	0.184
Living Alone	3.12	0.51	1.16	8.40	0.024	2.29	0.40	1.05	5.01	0.038
SRH (Poor)	0.99	0.45	0.41	2.37	0.975	1.51	0.41	0.67	3.37	0.317
Depression	1.01	0.08	0.86	1.19	0.879	1.14	0.08	0.98	1.32	0.086
Obesity	1.12	0.48	0.44	2.86	0.815	2.07	0.36	1.03	4.17	0.042
Constant	27.73	3.30	-	-	0.314	0.03	2.15	-	-	0.109
*Model 4*										
Age (Years)	0.96	0.04	0.90	1.03	0.248	1.06	0.02	1.01	1.11	0.010
Educational Attainment	0.90	0.12	0.71	1.14	0.361	1.02	0.10	0.84	1.25	0.837
Financial Difficulty	0.91	0.05	0.83	0.99	0.026	0.92	0.04	0.86	0.99	0.030
Monthly Income	0.92	0.19	0.63	1.33	0.643	0.82	0.20	0.56	1.19	0.293
Marital Status (Married)	4.01	0.88	0.72	22.42	0.114	1.91	0.92	0.32	11.57	0.481
Living Alone	2.99	0.59	0.94	9.57	0.065	2.00	0.42	0.88	4.56	0.099
SRH (Poor)	0.95	0.50	0.36	2.54	0.919	1.19	0.44	0.51	2.80	0.693
Depression	0.91	0.09	0.77	1.09	0.326	1.11	0.08	0.95	1.29	0.179
Obesity	0.99	0.53	0.35	2.81	0.986	1.68	0.38	0.80	3.52	0.170
Multimorbidity	2.03	0.18	1.43	2.89	0.000	1.56	0.12	1.22	1.99	0.000
Constant	21.98	3.64	-	-	0.396	0.01	2.32	-	-	0.035

CDs: Chronic Diseases; SRH: Self-Rated Health. Model 3: Obesity only. Model 4: Obesity and multimorbidity.
